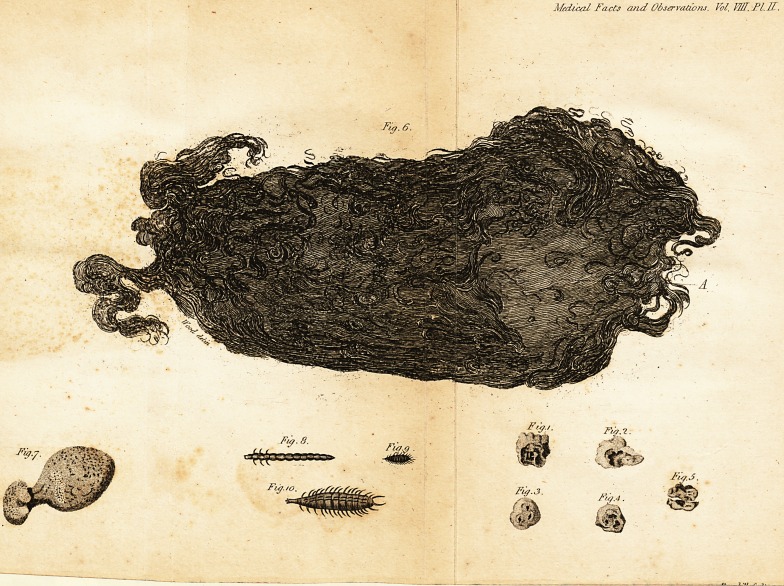# A Case of Hairy Concretions Found in the Human Stomach

**Published:** 1800

**Authors:** William Wood

**Affiliations:** Surgeon at Wingham, in Kent; and Fellow of the Linnean Society in London.


XIII. *A Caſe of hairy Concretions found in the human Stomach. By Mr.* William Wood, *Surgeon at Wingham, in Kent; and Fellow of the Linnean Society in London. Communicated in a Letter to* Samuel Foart Simmons, *M. D. F. R. S.*

*To Dr.* SIMMONS.

SIR,

HAVING lately received from my friend Mr. William Sankey, a reſpectable ſurgeon at Eythorne, in this neighbourhood, a very remarkable caſe of hairy concretions found in the human ſtomach; I take the liberty liberty of communicating it, for insertion, if you think it deserving of a place, in the Medical Facts and Observations.

Mr. Sankey's account of the case is as follows:

"Mary Spain, aged twenty-two years, "was always healthy till she was about eigh-"teen. She began to menstruate at the "age of twelve, and continued to do so, "without much irregularity, till she was eigh-"teen; at that age she became irregular, and "began to have symptoms of chlorosis, at-"tended with vomiting, constipation of the "bowels, and, sometimes, with most violent "pains, resembling the pains of labour. These "symptoms generally returned every three or "four months, and continued to do so till her "death. In the month of August, 1796, I "first saw her; her pains were then so violent, "and so much resembled labour pains, that I "really thought her in labour, particularly "as the menses were obstructed, and she had "a considerable swelling of the abdomen. In "a few days she became easier as usual, and "continued so for three or four months, at "which period she seldom failed to have a re-"turn of the symptoms. I did not see her "from that time till the 16th of November,

" 1797, when I found her in the ſame ſitua-

" tion as before. By the uſe of opiates, &c.

" for two or three days her pains were abated;

" but on December 2d ſhe ſent for me again,

" in conſequence of having voided a ſmall

" lump of hair the day before. She was now

" better, and began to be in hopes of getting

" well. On the 26th of December, the pain,

" vomiting, &c. returned, and on the next

" day ſhe died. On the 30th I opened the

" body. The abdomen was very much diſ-

" tended, and upon opening it, a large quan-

" tity of a dark coloured fœtid fluid was found

" extravaſated among the inteſtines. The me-

" ſentery was in a ſtate between ſuppura-

" tion and mortification. The ſmall inteſtines

" were much inflamed, and covered with that

" kind of matter which is ſaid to be found in

" thoſe who die of the puerperal fever. The

" whole of the canal was empty. The uterus,

" bladder, kidneys, liver, ſpleen and pancreas,

" were in a perfectly natural ſtate. The ſto-

" mach appeared ſound, both on the outſide

" and on the inſide, and contained nothing

" but the two lumps of hair, which you may

" ſuppoſe, by their ſize and ſhape, nearly

" filled it. The hair, in colour and texture,

" ſeems

"seems much like her own, which, when a

"child, used to be long, but lately her friends

"had taken notice that it was always short.

"This in some measure accounts for the hair

"in her stomach, though no person ever saw

"her swallow any."

Of the largest of the two masses of hair sent to me by Mr. Sankey, I have made a drawing, which accompanies this letter.

This is composed, like the others, of coarse black hair matted together, (with what I conjecture to be the aliment,) taken into the stomach at different times, and there, by the action of that organ, united into a mass. At the large end (See A. Fig. 6, Plate II.) is an excavation which may have been formed by that end lying near the œsophagus, exposed to the immediate action of all the fluid taken into the stomach. Round the edge of the lump, at different places, are locks of hair of considerable length.

The mass, next in size to that which I have just now described, assumes a different form, although it consists in like manner of cemented hair, two or three locks of which may be seen at the bottom of the mass. That I might be certain no calcareous matter entered into the composition compoſition of the lumps, a ſmall portion of them was put into a glaſs with ſome marine acid; but not the ſmalleſt efferveſcence was perceptible.

Of the two ſmaller maſſes, mentioned by Mr. Sankey, the leaſt was found in the ſtomach, and ſeems to be a fragment from a larger lump. The other was paſſed by the rectum, and ſtill retains the figure which it acquired in its paſſage.

The dimenſions and weights of theſe four different pieces are as follow:

### DIMENSIONS.

| | | INCHES. |
|---|---|---|
| No. 1, | Length . . . | 6 .. $\frac{1}{2}$ |
| | Breadth . . . | 3 .. $\frac{1}{2}$ |
| | Circumference . | 9 .. $\frac{7}{8}$ |
| No. 2, | Length . . . | 5 .. $\frac{5}{8}$ |
| | Breadth . . . | 3 .. $\frac{6}{8}$ |
| | Circumference . | 9 .. $\frac{3}{4}$ |
| No. 3, (The Fragment.) | Length . . . | 2 .. $\frac{3}{4}$ |
| | Breadth . . . | 1 .. $\frac{3}{4}$ |
| | Circumference . | 4 .. $\frac{1}{4}$ |

| | | INCHES. |
|---|---|---|
| No. 4, (The Piece paſſed by the Rectum.) | Length . . . | 3 .. $\frac{3}{4}$ |
| | Breadth . . . | 1 .. $\frac{5}{8}$ |
| | Circumference . | 4 .. $\frac{3}{8}$ |

WEIGHTS,

WEIGHTS, (in their prefent dry State)

| | oz. | dr. | gr. |
|---|---|---|---|
| No. 1, . . . . . | 5 | 0 | 27 |
| No. 2, . . . . . . . | 4 | 5 | 36 |
| No. 3, . . . . . . . | 0 | 3 | 40 |
| No. 4, . . . . | 0 | 3 | 37 |
| Total . | 10 | 5 | 20 Troy. |

Concretions of this kind are fometimes found to occur in cows, and other animals; but I had not been able to meet with any inftance of a fact, fimilar to the prefent, in the human body, till you pointed out to me one, which does indeed exhibit a very ftriking affinity to that which I have been relating. It was firft publifhed in the *Journal de Medecine* for December, 1779; and an abridged account of it, with engraved figures of the concretions, of their natural fize, was afterwards inferted in the *Hiftoire de la Societé Royale de Medecine,* (années 1777 & 1778,) publifhed at Paris in 1780. In your account of the latter work, in the London Medical Journal, (Vol. IV. Page 361) you have given a concife but accurate defcription of the cafe, which I fhall beg leave to tranfcribe:

" Defcription

*Fig. 6.*

*Fig. 7.*

*Fig. 8.*

*Fig. 9.*

*Fig. 10.*

*Fig. 1.*

*Fig. 2.*

*Fig. 3.*

*Fig. 4.*

*Fig. 5.*

" Defcription of two maffes of hair, found in
" the ftomach and bowels of a boy, fixteen
" years old. By M. Baudamont, furgeon
" at Verdun.

" The patient, who is the fubject of this " hiftory, had from his infancy delighted in " fwallowing hairs; fo that he not only pluck-" ed his own, but likewife thofe of his brother, " and others. This fingular tafte increafed " as he grew up. The hairs, thus fwallowed, " formed an oval mafs, which might be felt " a little below the pit of his ftomach. At " length his ftomach became painful; he had " diarrhœa; frequent and violent vomiting; " and fever, which gradually terminated in " death. On diffection, two maffes of hair " were difcovered. The largeft of thefe com-" pletely filled the ftomach, and extended " throughout the duodenum. This had a " cherry ftone for its nucleus. The other " fmaller mafs was in the jejunum. The two, " when recent, were moiftened with a very " fœtid mucus, and weighed two pounds " and an ounce. In a dried ftate, their

"weight was reduced to eleven ounces and a
"half."

## Figures and Tables

**Fig.1. Fig.2. Fig.3. Fig.4. Fig.5. Fig.6. Fig.7. Fig.8. Fig.9. Fig.10. f1:**